# The Staffordshire Arthritis, Musculoskeletal, and Back Assessment (SAMBA) Study: a prospective observational study of patient outcome following referral to a primary-secondary care musculoskeletal interface service

**DOI:** 10.1186/1471-2474-11-67

**Published:** 2010-04-08

**Authors:** Edward Roddy, Irena Zwierska, Peter Dawes, Samantha L Hider, Kelvin P Jordan, Jon Packham, Kay Stevenson, Elaine Hay, the SAMBA team

**Affiliations:** 1 Arthritis Research Campaign National Primary Care Centre, Primary Care Sciences, Keele University, Staffordshire, ST5 5BG, UK; 2 Staffordshire Rheumatology Centre, Haywood Hospital, High Lane, Burslem, Stoke-on-Trent, ST6 7AG, UK; 3 University Hospital of North Staffordshire, London Road, Stoke-on-Trent, ST4 6QG, UK

## Abstract

**Background:**

Recent healthcare policy has shifted the management of musculoskeletal conditions in the UK away from secondary care towards Clinical Assessment and Treatment Services at the primary-secondary care interface. However, little is known about the outcome of patients with musculoskeletal conditions referred from primary care to Clinical Assessment and Treatment Services or how best to identify those patients at high risk of poor outcome in this setting. We describe the protocol for a twelve-month prospective observational study which aims to describe the outcome of patients referred to musculoskeletal and back pain services at the primary-secondary care interface and to develop simple prognostic measures to guide clinical prioritisation and triage.

**Methods/Design:**

All patients referred over a twelve-month period from primary care to musculoskeletal and back pain clinics in the primary-secondary care interface Clinical Assessment and Treatment Service in North Staffordshire will be mailed a postal questionnaire prior to their consultation. This will collect information on quality of life, general health, anxiety and depression, pain, healthcare utilisation including medication use, occupational characteristics, and socio-demographics. At the consultation in the interface clinic, the clinical diagnosis, investigations requested, and clinical interventions will be recorded. Follow-up data for the twelve-month period subsequent to recruitment will be collected via mailed follow-up questionnaires at 6 and 12 months, and review of medical records.

**Discussion:**

This twelve-month prospective observational study of patients referred to a musculoskeletal Clinical Assessment and Treatment Service will assess the management and outcome of musculoskeletal care at the primary-secondary care interface as proposed in the Musculoskeletal Services Framework.

## Background

Musculoskeletal conditions such as back pain, osteoarthritis and regional pain are highly prevalent and associated with a considerable burden of pain, disability and work loss [1]. It is estimated that one in five adults will consult primary care for a musculoskeletal problem during a one-year period [2]. Most non-inflammatory musculoskeletal disorders are managed in primary care. Traditionally, referral to secondary care has been to specialist services such as orthopaedics or rheumatology, in a setting supported by the multidisciplinary team. Whilst certain patients referred to secondary care require care in that setting, particularly those deemed suitable for joint replacement surgery, many patients can be managed appropriately at the interface between primary and secondary care. The recently published Musculoskeletal Services Framework [3] and UK Government White Paper "Our Health, Our Care, Our Say" [4] outline a change in emphasis shifting the management of musculoskeletal conditions in the UK away from secondary care towards Clinical Assessment and Treatment Services (CATS) at the primary-secondary care interface. This change aims to improve efficiency, provide treatment closer to patients' homes, and, with effective triage, reduce inappropriate referrals to secondary care services such as rheumatology and, in particular, orthopaedics [5]. The generic functions of CATS are to provide an expert multidisciplinary opinion, screen for important remedial conditions and refer such conditions appropriately, direct patients to appropriate services for investigation, agree evidence-based integrated care pathways, facilitate referral to other primary and secondary care services where necessary, and support the development of robust systems for monitoring and clinical audit [3].

The Musculoskeletal Services Framework identified several key issues with traditional referral pathways from primary to secondary care, including long waiting times for assessment and treatment which are unacceptable to patients [6] and may adversely influence outcome [7,8], and a lack of clear integrated care pathways [3]. It attempts to tackle these issues by proposing the development of pathways which manage patient flows through primary/secondary care to ensure appropriate and timely referral to specialist care services and use capacity in acute settings appropriately. Effective triage to ensure patient referrals from primary care reach the most appropriate destination is a key component of such pathways. Traditionally, both the triage destination and clinical priority have been determined by the information provided in the referral letter. However, many referrals to hospital-based musculoskeletal services are misdirected and provide insufficient clinical information to guide triage [9]. The Musculoskeletal Services Framework has proposed use of tools to guide clinical triage and prioritise urgency of surgery [10-12]. However, existing measures are joint-specific and have tended to focus on surgical priority in secondary care. There is a need for generic clinical priority measures, which can be used to guide clinical triage across a wider range of musculoskeletal conditions at the primary-secondary care interface.

Musculoskeletal conditions which might previously have been managed in secondary care will be increasingly managed at the primary-secondary interface. However, there is a paucity of evidence on the transition of patients from primary to secondary care and outcome of patients with musculoskeletal conditions referred from primary care to CATS. The natural history, progression and outcome in patients with a range of musculoskeletal conditions have been described in primary care [13-17] and secondary care (i.e. rheumatology) [18-20] settings. A small number of studies have examined the outcome of GP referrals to secondary care (including orthopaedic and rheumatology clinics) and have highlighted issues such as high rates of re-attendance [21], the poor outcome of many patients [22,23] and the role of patient pressure in the referral process [24]. These studies have tended to focus on a single or limited number of disease areas, for example, knee osteoarthritis, shoulder pain or rheumatoid arthritis.

**Figure 1:**
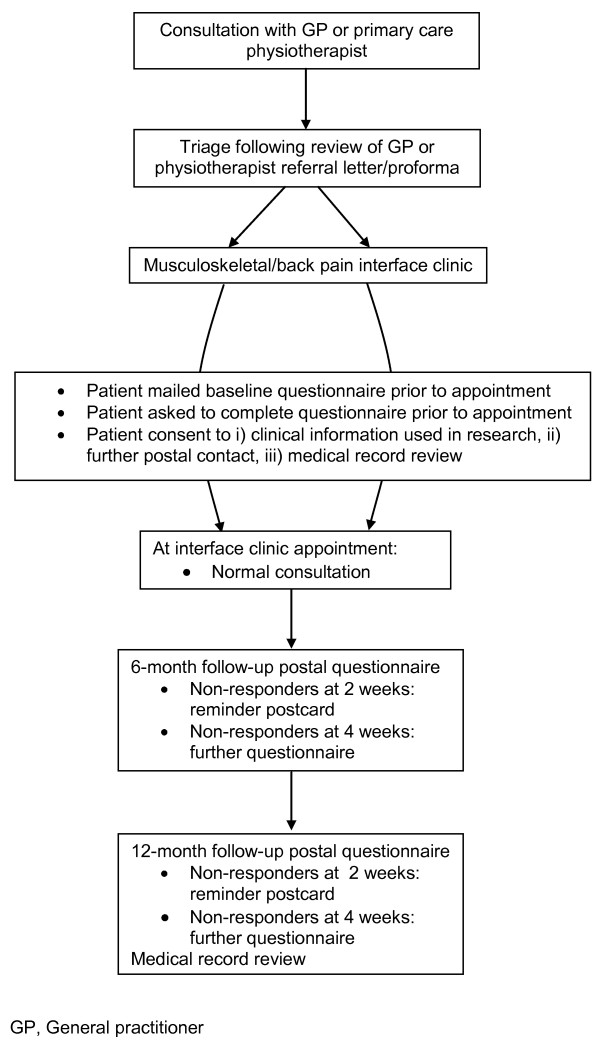
**Flow diagram outlining participant's study pathway**.

We describe here the protocol for a twelve-month prospective, observational cohort study which aims, firstly, to describe the characteristics, management, and clinical and health-economic outcomes, of patients with a wide range of musculoskeletal conditions referred from primary care to an established musculoskeletal interface CATS, and, secondly, to develop simple prognostic measures for use as clinical triage tools to identify patients at high risk of poor outcome from musculoskeletal conditions and back pain at the primary-secondary interface (Figure 1).

## Methods/design

### Design

The study will be a twelve-month prospective observational study undertaken in the setting of musculoskeletal interface services in North Staffordshire. Ethical approval for the study has been obtained from South Staffordshire Local Research Ethics Committee (REC reference number: 07/H1203/86).

### Study setting and population

Musculoskeletal interface services in North Staffordshire were highlighted in the Musculoskeletal Services Framework as a successful model of interface care which incorporate dynamic triage of pooled musculoskeletal referrals and clinical algorithms for a wide range of conditions. CATS clinics are located in local hospitals, health centres and general practices [3]. These CATS clinics utilise multidisciplinary staffing including rheumatologists, consultant physiotherapists, extended scope physiotherapists and GPs with special interests. The service has reduced waiting times in accordance with current NHS targets, increased orthopaedic conversion rates and been highly rated in satisfaction surveys of patients and GPs [3].

The study population will consist of all patients referred from primary care and subsequently triaged to musculoskeletal and back pain interface clinics in Stoke-on-Trent Primary Care Trust (PCT) over a twelve-month period. All adults aged 18 years and over and able to understand and capable of giving written informed consent will be considered eligible to participate in the study.

### Initial contact

Referral letters for all patients with musculoskeletal problems will be triaged by the clinical team according to usual clinical care. Those patients triaged to the CATS will be potentially eligible for inclusion in the study and will be sent a study pack two weeks prior to their appointment containing a letter of invitation, study participant information sheet and baseline self-administered questionnaire consisting of validated health assessment instruments. The appointments process will generate a study database to facilitate mailing of study packs directly to patients. Patients will be asked to complete the baseline questionnaire prior to their appointment but will also be advised that the study will be explained to them again when they attend for the clinic appointment, so that they can ask any questions they wish at that time. Patients will be informed that there is no obligation for them to participate if they do not wish to do so, and if they decline they will continue to receive normal clinical care. Patients willing to participate will be asked to provide full written informed consent. The last page of the questionnaire will include three consent questions; (1) for use of clinical information taken at their initial clinic visit to be used in the research, (2) for further contact by postal questionnaire; (3) for review of medical records.

### Baseline questionnaire

Details of the information to be collected by the baseline questionnaire are presented in Table 1. This includes validated quality of life, general health, musculoskeletal-related, demographic, occupational and health care use scales. It will also include three potential prognostic tools: the modified Salisbury score is a clinical priority score used in routine clinical practice in Stoke-on-Trent (table 2) [25,26]. We have developed a patient-completed version of the Salisbury score, which we aim to validate in this study. The PROG-RES [27] and StarT Back tools [28] have been developed by our centre with the aims of identifying patients at high risk of poor outcome from musculoskeletal conditions and back pain in primary care.

**Table 1:** Content of the baseline postal questionnaire

Concept	Measurement method	Detail
Quality of life	EuroQOL (EQ-5D) [31]	Five dimension descriptive system (today)
Perceived general health	MOS Short Form-36 (SF-36) version 2 [32]	36-items covering eight health domains (past 4 weeks)
Anxiety and depression	Hospital anxiety and depression scale [33]	Anxiety and depression sub-scales (past week)
	Depression Screening questions for primary care [34]	Low mood/anhedonia in the last month
Bodily pain	Location: Self-completed manikin	"In the past 4 weeks, have you had pain that has lasted for one day or longer in any part of your body?"
	Current pain severity [35]	0-10 NRS with verbal anchors (no pain, pain as bad as can be)
	Episode duration [36]	<3 months, 3-6 months, 7-12 months, 1-2 years, 3-5 years, 6-10 years, 10+ years
Clinical triage and prioritisation tools	Patient-completed Salisbury score	6-item triage tool for musculoskeletal problems referred from primary care
	PROG-RES tool [27]	7-item prognostic tool for musculoskeletal pain in primary care
	Modified STarT Back screening tool [28]	7-item modified screening tool for musculoskeletal prognostic indicators including 5-item psychosocial subscale (past 2 weeks)
Knee pain	Western Ontario and McMaster Universities Osteoarthritis index (WOMAC) [37]	Pain (0-20), stiffness (0-8), physical functioning (0-68)
Healthcare utilisation for musculoskeletal problems	Consultations	GP, hospital doctor, practice nurse, district nurse, physiotherapist, osteopath, other
	Investigations/treatment	
	Medication use	Analgesics, NSAIDs, opiates, natural remedies, glucosamine, chondroitin
Occupational characteristics	Job title/type of work for most of working life	
	Current employment status	Working full-time/part-time, Employed but off-sick for < 6 months, Looking after home/children, Not working for > 6 months due to joint/back problem, Fully retired, Early retirement due to joint/back problem, Student
	Work absence during last 6 months due to joint/back problems	Yes/No
	Current work status	Doing usual job, working fewer hours, doing lighter duties, paid/unpaid sick leave
	Stanford presenteeism scale (SPS-6) [38]	6-items concerning Health Status and Employee Productivity (5-point Likert scale)
	Effect of joint/back problems on productivity	0-10 NRS with verbal anchors (no effect, completely prevented me from working)
Demographic characteristics	Date of birth, gender	
	Marital status	Married, separated, divorced, widowed, cohabiting, single
	Living arrangements	Alone, not alone
Anthropometric data	Self-reported height	
	Self-reported weight	
Lifestyle characteristics	Smoking status	Never, previously, currently
	Alcohol intake	Daily, weekly, monthly, yearly, never

**Table 2:** Modified Salisbury score completed by referring GP/physiotherapist and interface-clinician

**Please rate your assessment of the patient's current pain/problems:**		
		
1. How do you rate the progress of the problem?	2. How do you rate the pain that the patient is experiencing?	3. How do you rate the distress that the patient is experiencing (psychosocial)?
		
Stable/improving (0)	No pain (0)	None (no worry) (0)
Slowly worsening (months) (1)	Occasional pain (1)	Mild (occasional worry) (1)
Worsening steadily (weeks) (2)	Frequent pain (2)	Moderate (frequently worried (2)
Rapidly worsening (days) (3)	Constant pain (night & day) (3)	Severe (constant distraction) (3)
		
4. How do you rate the loss of physical function?	5. How do you rate the patient's dependence on others?	6. How do you rate the specific effect on the patient's ability to perform normal activities during the last week (ie social, housework, educational, recreational)?
		
0-25% loss of function (0)	No dependence (0)	Not affected (0)
26-50% loss of function (1)	Occasional help needed (1)	Coping but affected (1)
51-75% loss of function (2)	Regular help needed (2)	Not coping some days (<3 days) (2)
76-100% loss of function (3)	Substantial dependence (3)	Total incapacity (3)

### Clinic appointment

When they attend for their routine clinic appointment, patients will be seen by a research assistant who will explain the study again, answer any questions the patient may have, and check the questionnaire and consent form for completeness. The clinical diagnosis, investigation(s) requested, intervention(s), and plan for clinical follow-up will be recorded on a standard paper proforma by the interface clinician conducting the clinical consultation. The interface clinician will also be asked to complete the modified Salisbury score (Table 2). This score is also routinely completed by the referring GP or physiotherapist at the time of referral to musculoskeletal services at the primary-secondary care interface. The referrer-completed modified Salisbury score will be transposed from the referral letter (where completed).

### Pilot of baseline procedure

The self-report questionnaire will be piloted in a small number of patients attending two musculoskeletal clinics to assess its acceptability to patients and ease of completion (approximately 5-10 patients). The processes for recruitment and obtaining consent will be piloted in one back pain and one musculoskeletal clinic per week over a one-month period (approximately 100 patients). The clinician paper proforma was developed in consultation with musculoskeletal interface clinicians and piloted in CATS clinics.

### Follow-up questionnaires

A self-administered questionnaire will be mailed to all participants at 6 and 12 months. Non-responders will be sent a postcard reminder after 2 weeks and a repeat questionnaire after 4 weeks. The 6- and 12-month follow-up questionnaires will be identical to the baseline questionnaire (Table 1). Participants will also be asked to rate (1) their satisfaction with the care they have received on a 5-point Likert scale ranging from "Very satisfied" to "Not at all satisfied" and (2) how their musculoskeletal problem compares to 6 months previously on a 5-point Likert scale ranging from "Much better" to "Much worse".

### Medical record review

Medical record review from baseline to 12 months will seek to identify: (1) relevant comorbidities, (2) repeat consultation in primary care with the same musculoskeletal problem (clinician-recorded diagnosis mapped to standard Read morbidity codes [29]) and, (3) further referral to interface clinics or secondary care.

### Sample size

Approximately 3500 patients are seen in the musculoskeletal and back pain interface clinics in North Staffordshire during the course of a year. Based on previous studies, we would expect 75% of these to participate in the baseline stage (*n *= 2625). Based on previous work within our Centre [30], we would expect 75% of these to consent to further contact and 75% to consent to medical record review. This would mean around 2000 participants having their medical records reviewed. Given a 75% response to each follow-up questionnaire, then around 1500 people would return 6-month questionnaires and 1125 people 12-month questionnaires. A sample size of 1125 is sufficient to determine the percentage referred to the interface clinics who make a repeat consultation to primary care during the course of the following year with a margin of error of 3% and a 95% confidence level, based on an actual estimate of 50%.

### Analysis

The primary analysis will be simple descriptive statistics (frequencies, percentages, means, medians where appropriate with confidence intervals) of the characteristics of patients seen in interface clinics at baseline, and the number of repeat consultations to primary care over the twelve-month period. Changes within outcome measures (e.g. EuroQOL, Short Form-36 (SF-36), Hospital Anxiety and Depression Scale) over the duration of the study will also be examined using paired t-tests for continuous data and McNemar tests for dichotomous data.

Secondary analyses will involve comparisons between different patient groups (depending on the case-mix seen) and to determine baseline factors predicting change on outcome measures over the course of the study and clinical response to specific interventions. Multiple linear regression will be used for continuous outcomes and logistic regression for dichotomous outcomes.

The three modified Salisbury scores (referrer-completed, musculoskeletal clinician-completed and patient-completed) will be compared to each other at baseline using intraclass correlation coefficients. Each modified Salisbury score will be compared to the primary care musculoskeletal assessment tool [27] and STarT Back screening tool [28] using Pearson's correlation coefficient at baseline. The ability of each to predict repeat consultation, global change and changes within other health status measures (e.g. SF-36) over the duration of the study will be examined by comparing those with predicted poor outcomes to those with better prognoses on the prognostic tools. Sensitivity and specificity measures will be used to assess prognostic ability compared to the outcome measures.

## Discussion

Recent UK healthcare policy has advocated providing non-surgical care of common musculoskeletal conditions such as back pain, osteoarthritis and regional pain in CATS at the primary-secondary care interface [3]. However, a paucity of research evidence exists to support this policy and the clinical and cost-effectiveness of such interface services is not known. This protocol describes a twelve-month prospective observational study of the characteristics, management and outcome of patients referred to a musculoskeletal CATS at the primary-secondary interface in North Staffordshire. The study will assess the effectiveness of musculoskeletal care at the primary-secondary care interface, determine simple tools to guide prioritisation, and potentially influence the design of future care pathways.

## Competing interests

The authors declare that they have no competing interests.

## Authors' contributions

All authors participated in the design of the study, acquisition of data, and drafting of the manuscript. All authors read and approved the final manuscript.

## Pre-publication history

The pre-publication history for this paper can be accessed here:

http://www.biomedcentral.com/1471-2474/11/67/prepub
